# Socio-ecological model: Understanding facilitators and barriers to HIV testing and counseling uptake in sub-Saharan Africa – A systematic review

**DOI:** 10.1016/j.dialog.2025.100273

**Published:** 2025-12-23

**Authors:** Amanda Debuo Der, Elvis Enowbeyang Tarkang

**Affiliations:** a Department of Population, Family and Reproductive Health, School of Public Health, University of Ghana, Ghana; b School of Public Health, University of Health and Allied Sciences, Ho, Volta Region, Ghana; c HIV/AIDS Prevention Research Network Cameroon, Kumba, Southwest Region, Cameroon; d School of Nursing and Public Health, University of KwaZulu-Natal, Durban, South Africa

**Keywords:** HIV testing and counseling, Socio-ecological model, Africa, Sub-Saharan Africa

## Abstract

**Objective:**

This systematic review examined the facilitators and barriers to HTC uptake among adults in SSA using the socio-ecological model (SEM).

**Methods:**

A systematic search of Google Scholar, PubMed, Scopus, Sociological Abstracts, PsycINFO and Europe PMC identified 114 HTC uptake studies in SSA from 2010 to 2023 of which 6 met the inclusion criteria. The search followed the Preferred Reporting Items for Systematic Reviews and Meta-Analyses (PRISMA) guidelines.

**Results:**

Individual level facilitators of HTC included high perceived HIV risk, privacy, higher education, and prior testing experience while low HIV knowledge, fear of positive results, stigma, and lack of confidence in self-testing were barriers. Interpersonal facilitators included partner and peer support, and peer normalization of testing, while barriers involved fear of partner reactions, trust concerns, and fear of unintentional disclosure. At the health system-level, facilitators included counseling, positive experiences with providers, and no discrimination while privacy concerns, stigma, and judgmental attitudes of healthcare workers hindered HTC uptake. The community-level barriers included misconceptions and stigma, whereas community-based education improved HTC uptake. At the policy level, cost was a barrier, but availability of self-testing facilitated uptake. The interpersonal factors presented the most significant facilitators (53.3 %), while the policy-level factors were the most significant barriers (57.1 %).

**Conclusion:**

HTC uptake in SSA is shaped by intersecting SEM factors. Understanding these influences are essential for designing effective interventions to improve HTC uptake and linkage to care.

**PROSPERO ID:** CRD42024583105.

## Highlights

• Critical gaps in HIV prevention impede achievement of 95–95-95 goals.
• Interpersonal support strongly boosts HIV testing uptake in Sub-Saharan Africa.
• Community education reduces stigma and increases HIV testing participation.
• Fear of partner reaction is a major barrier to voluntary HIV testing uptake.
• Supportive healthcare settings encourage HIV testing and counseling.

## 1 Introduction

The Millennium Development Goal (MDG) 6, which targeted the reduction of the spread of the Human Immunodeficiency Virus (HIV)/Acquired Immune Deficiency Syndrome (AIDS) by 2015 [1], was not achieved but new infections have been gradually declining [2]. Notwithstanding this, there is still a window of opportunity to meet Sustainable Development Goal 3 (SDG 3), which is focused on guaranteeing health and wellness for all ages [3]. This study also aligns with the World Health Organization's [WHO] Global Health Sector Strategy on HIV 2022–2030, which aims to reduce new HIV infections, expand access to HIV testing and treatment, and eliminate HIV-related stigma and discrimination [4]. HIV testing and counseling (HTC) is a crucial prevention strategy in the reduction of HIV/AIDS. Despite the gains recorded in reducing new cases of HIV/AIDS worldwide, some countries in sub-Saharan Africa (SSA) still have the highest incidence [5]. At the end of 2022, 39 million people were living with HIV globally with varying burdens between countries and regions with 1.3 million new infections [6]. The WHO African region is severely affected and accounts for two-thirds of the people living with HIV with a rate of at least 1 in every 25 adults [6].

To address the HIV burden, the Joint United Nations Programme on HIV/AIDS (UNAIDS) established the 95–95-95 targets; 95 % of persons living with HIV (PLHIV) should know their status, 95 % of those diagnosed should be receiving antiretroviral therapy (ART) and 95 % of those receiving treatment should achieve viral suppression [7]. Progress toward the achievement of these goals is vital in SSA considering the HIV burden. So far, only five SSA countries (Rwanda, Botswana, Eswatini, United Republic of Tanzania, and Zimbabwe) achieved the 95–95-95 targets by 2022 [8]. Though the UNAIDS reported a decline in HIV new infections in SSA in 2023, there is a need to understand the drivers of HTC uptake to accelerate the achievement of the targets in the remaining SSA countries.

**Fig. 1:**
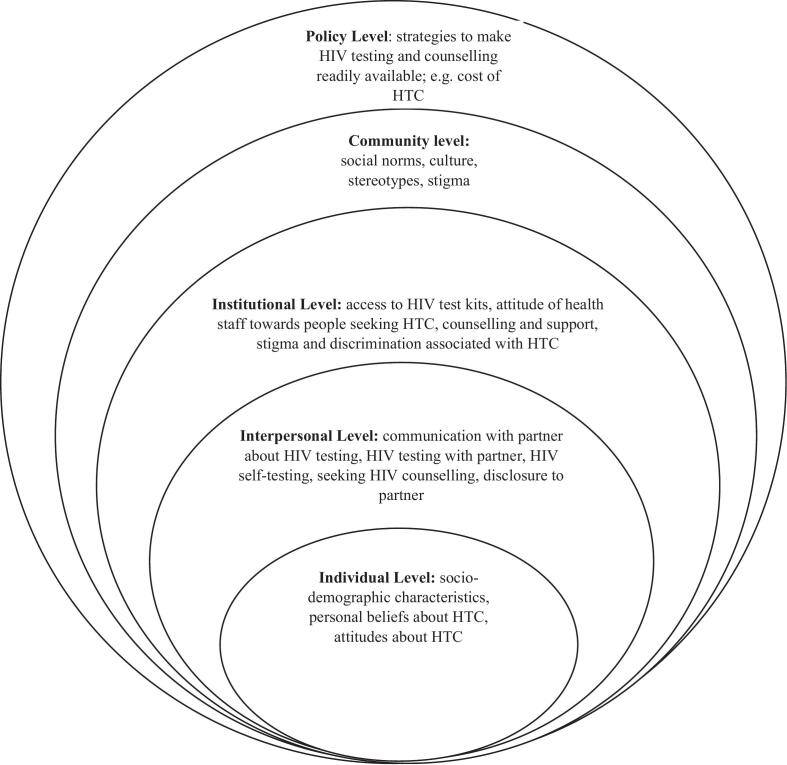
The socio-ecological model (adapted from McLeroy et al., 1988).

The socio-ecological model (SEM) is essential for understanding the multifaceted and interactive effects of personal, interpersonal, community, institutional, and policy-level factors that affect behavior and leveraging these to improve health behavior [[9], [10], [11]]. The SEM has been used extensively in both qualitative and quantitative research in understanding and planning health interventions such as intimate partner violence and HIV infection in the Caribbean [12] barriers to accessing HIV care in Southern Malawi [13], HIV testing in Kenya [14], HIV testing in a clinic-based setting in South Africa [15], and HIV-related sexual risk behaviors among young people in Kenya [16]. Given that HTC uptake determinants are multi-faceted, this systematic review sought to understand HTC uptake based on the SEM (Fig. 1). This aggregation of literature provides a vivid view of where HTC programs in SSA should be targeted to ensure the acceleration of efforts to meet the global goals.

## 2 Methods

### 2.1 Study design

This study is a systematic review of published articles on HTC uptake that used the SEM as the framework. The methodological framework used is according to the five-stage outline described by Arksey and O'Malley [17]. Stage one (1) involved identifying a research question; stage two (2) encompassed identifying relevant studies; stage three (3) involved the study selection; stage four (4) involved charting of the data and stage five (5) encompassed collating, summarising and reporting the results. The systematic review protocol has been registered in PROSPERO **(registration ID: CRD42024583105).** The protocol can be accessed at PROSPERO.

### 2.2 Search strategy and article selection

**Fig. 2:**
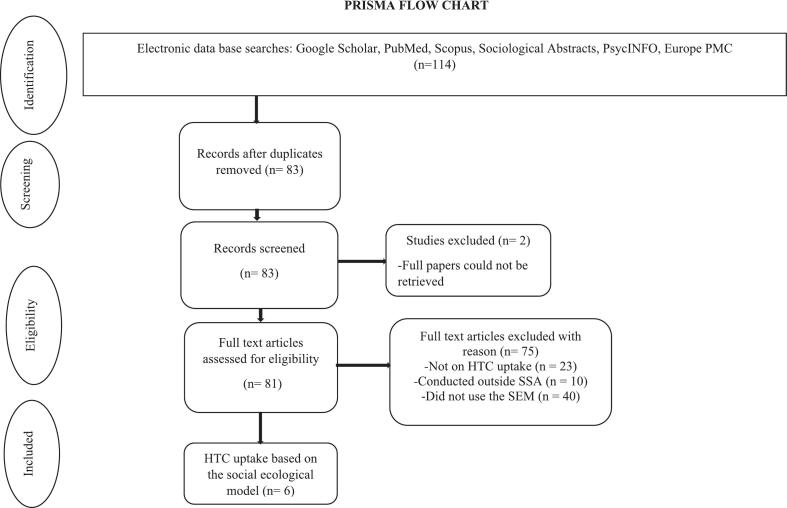
PRISMA flowchart showing the process of the review.

A systematic search was conducted in Google Scholar, PubMed, Scopus, Sociological Abstracts, PsycINFO, and Europe PMC for articles that met the eligibility criteria set by the authors. The search sought studies that were published on the SEM and HTC uptake in SSA from 2010 to 2023. The search followed the Preferred Reporting Items for Systematic Reviews and Meta-Analyses (PRISMA) guidelines (Fig. 2). The following terms were used (including synonyms and closely related words) as index terms or free-text words: “HIV testing and counseling” “Sub-Saharan Africa”, “Socio-ecological model” and “HIV testing and counseling AND Socio-ecological model”. Further, Medical Subject Headings (MeSH terms) such as “HIV testing,” “HIV testing and Counseling,” and “Sub-Saharan Africa” were used to enhance the search strategy by standardising terminology, ensuring comprehensive retrieval of relevant studies, and reducing the risk of missing important articles due to variations in wording or terminology. Also, English was the only language used for the search. Articles published in languages without translation and titles for which the complete text could not be retrieved were excluded from this review. All citations were exported into EndNote Version 20 and duplicate citations were manually removed. Other duplicates were removed during the screening and data extraction process. The search, revision of the articles and extraction took place from August – December 2023. Articles published on HTC uptake without the use of the SEM as a framework were excluded. This ensured that the categorisation of facilitators and barriers at the various levels (individual, interpersonal, community, institutional, and policy) was grounded in the authors' intended theoretical framing rather than inferred subjective interpretation and categorisation of the results. This helped prevent the introduction of bias and maintained consistency in how constructs were defined and interpreted across studies.

The selection of the studies was done independently by the two authors. The selection considered a number of other factors. First, the reliability of the studies (whether the effect size was enough, and the findings reported with the confidence levels for quantitative studies). Also, the relevance of the studies was checked to see if the research methodology used was appropriate for answering the research question. Further, the appropriateness of the number of study participants in relation to the study design was considered to ensure that the results were not due to random variation.

For quality assessment, we applied established tools tailored to study design. For cross-sectional studies, we used key elements from the Joanna Briggs Institute (JBI) Checklist for Analytical Cross-Sectional Studies, including assessment of study design appropriateness, inclusion criteria, measurement validity, handling and adjustment for confounders, and statistical analysis [18]. For qualitative studies, we assessed congruity between methodology and research objectives, study design appropriateness, data collection methods and rigor, analysis, representation of participants' voices, and researcher reflexivity based on JBI qualitative appraisal guidance [19]. The summary of the appraisal outcomes, including JBI criteria met, key strengths, identified limitations, and overall quality ratings, are presented in Table 1. No study was excluded based on quality alone; however, the findings of studies with identified limitations were interpreted with caution. Any disagreements between reviewers during quality assessment were resolved through discussion and consensus.

**Table 1:** Quality assessment of included studies using JBI critical appraisal checklists.

Author, Year	Study Design	JBI Criteria Met	Key Strengths	Identified Limitations	Overall Quality
Christian et al., 2020	Qualitative	7/8	Methodological congruity; reflexivity addressed	Limited discussion of sampling strategy	High
Mendy et al., 2023	Cross-sectional	6/8	Appropriate analysis; moderate sample	Some confounders not fully adjusted	Moderate
Nnko et al., 2020	Qualitative	8/8	Clear congruence between philosophical perspective, methodology, data collection, and interpretation; reflexivity; clear methodology	No major issues identified	High
Kirakoya-Samadoulougou et al., 2017	Cross-sectional	7/8	Large sample; adjusted for confounders; valid measurement	Limited discussion on potential selection bias	High
Yakasai & Yakasai, 2022	Cross-sectional	7/8	Adjustment for confounders; representative sample	Potential recall bias	High
Okal et al., 2020	Qualitative	6/8	Data collection rigor; analysis appropriate	Minimal discussion of bias	Moderate

### 2.3 Data extraction

Both authors were involved in the selection of the studies and data extraction process. All full-text articles that were judged to be relevant to the study were thoroughly reviewed to extract the necessary information. A literature review table was used to extract information such as the authors of the study, year of publication, objective of the study, study site (country), study population, study design, and key findings of the study in relation to the levels of the SEM. The literature review table was piloted and revised to ensure that all the relevant information was captured. This enabled the authors to extract all relevant information from the 6 studies that met the final inclusion criteria. All included articles were renamed and saved in a separate folder to ensure easy tracking and revision.

## 3 Results

The findings from the studies included in the review were themed based on the levels of the SEM; individual, interpersonal, community, healthcare system or institutional, and the policy level factors that influence HTC uptake among adults in SSA.

### 3.1 Characteristics of the studies

Six studies were included in this review. Of these, 2 were conducted in Kenya with sample sizes of 24 and 38 respectively [14,20]; 1 study in the Gambia using 305 participants [21]; 1 study in Tanzania (23 in-depth interviews and 227 participatory group discussions) [22]; 1 study in Burkina Faso using national Demographic and Health Survey (DHS) data [23] and 1 study in Nigeria using the 2013 national DHS data of women within 15–45 years [24]. Three studies were qualitative inquiries [14,20,22], and the remaining were cross-sectional studies [21,23,24] (Table 2).

**Table 2:** Characteristics of the studies included in the review.

Authors and Year of Publication	Country	Study design	Sample size	Study population	Key Findings
Christian et al. (2020)	Kenya	Qualitative	24	Truck drivers	**Individual Level Factors** ***Barriers*** • Lack of confidence in administering the test and interpreting results • Fear of making mistakes and obtaining false results • Distrust in the efficacy of oral self-tests (e.g., belief that saliva cannot detect HIV) • Preference for healthcare provider-led testing • Perception that self-testing is culturally or racially inappropriate • Lack of time to visit clinics for testing or picking up self-test kits ***Facilitators*** • Desire for privacy and avoidance of stigma associated with clinic-based testing • Perception of self-testing as time-saving and convenient • Prior experience with HIV testing enhances confidence in self-testing • Reduced fear of pain compared to blood tests. **Interpersonal Level** ***Barriers*** • Fear of partner's reaction to test results, including violence or relationship breakdown • Concerns about partner trust ***Facilitators*** • Willingness to test with partners to learn each other's status • Support from partners to pursue testing and follow-up care • Open communication with partners and family members about HIV testing **Institutional Level Factors** ***Barriers*** • Concerns about privacy during clinic visits • Stigma at the clinic settings ***Facilitators*** • Positive past experiences with healthcare providers, including supportive and respectful interactions. • Desire for post-test counseling, preferably in person. • Trust and confidence in healthcare workers' and facilities • Health facilities are free of discrimination **Policy Level factors** ***Barriers*** • High cost of self-test kits (perceived as unaffordable by participants) • Lack of incentives for HIV testing ***Facilitators*** • Availability of HIV tests at both public and private facilities
Mendy et al. (2023)	The Gambia	Institutional-based Cross-sectional	305	Student Nurses and Midwives	**Individual Level Factors** ***Barriers*** • Fear of receiving an HIV-positive result and its associated stigma • Belief that HIV testing is unnecessary for those who feel healthy • Preference for self-testing (concerns about personal privacy and reluctance to disclose personal behaviors) ***Facilitators*** • Increased perceived risk of HIV due to personal behaviors • Awareness of the importance of knowing HIV status for health management **Interpersonal Level factors** ***Barriers*** • Concerns about how partners and peers might react to a positive result ***Facilitators*** • Supportive attitudes from friends and partners encouraging HIV testing • Peer acceptance and normalization of testing behavior in student groups **Institutional Level Factors** ***Barriers*** • Lack of adequate privacy during HIV testing in health facilities. • Perceived judgmental attitudes of healthcare workers ***Facilitators*** • Availability of counseling services and perceived confidentiality in health systems • Positive past experiences and attitudes from healthcare workers toward HIV counseling **Community Level Factors** ***Barriers*** • Stigma associated with HIV testing in rural communities • Misconceptions and stereotypes about individuals who seek HIV testing, e.g., assumptions of immoral behavior ***Facilitators*** • Community-based education programs to reduce stigma and promote testing • Outreach programs to raise awareness about HIV prevention and testing services
Nnko et al. (2020)	Tanzania	Qualitative	23 IDIs and 227 for Participatory Group Discussion	Female sex workers	**Individual Level Factors** ***Barriers*** • Lack of confidence in performing and interpreting self-tests accurately • Fear of receiving a reactive test result leading to distress or self-harm ***Facilitators*** • Desire for privacy and confidentiality in testing • Autonomy in managing one's health **Interpersonal Level Factors** ***Barriers*** • Fear of unintentional disclosure to partners, peers, or family members • Concerns about trust and misuse of test results in relationships **Institutional Level Factors** ***Barriers*** • Perceived or actual stigma in health facilities leading to fears of confidentiality breaches • Absence of post-test counseling **Community Level Factors** ***Barriers*** • Stigma associated with HIV and sex work hindering testing • Adverse social norms leading to reluctance to self-test or disclose results ***Facilitators*** • Community-based education and awareness programs to reduce stigma **Policy Level factors** ***Barriers*** • High cost of self-testing kits ***Facilitators*** • Availability of HIV self-testing for improved privacy
Kirakoya-Samadoulougou et al. (2017)	Burkina- Faso	Cross-sectional	Women = 14,656 Men = 5680	Demographic and Health Survey (DHS)	**Individual-Level Factors** ***Barriers*** • Respondents with poor knowledge of HIV transmission and prevention were less likely to be tested • Residing in rural areas decreased the likelihood of testing ***Facilitators*** • Participants with primary, secondary, or higher education were significantly more likely to be tested • Belonging to the higher wealth quintiles increased testing likelihood • High exposure to radio, television, and print media increased awareness and testing uptake • Accepting attitudes toward people living with HIV facilitated testing • Number of children ever born was significantly associated with HIV testing • Number of lifetime sexual partners • Belonging to the Christianity religion **Institutional Level Factors** ***Barriers*** • Limited accessibility to HIV testing services in rural areas. • Low availability of information about testing locations. ***Facilitators*** • Integration of HIV testing into antenatal care for women increased testing uptake. • No discrimination at the health facilities **Community-Level Factors** ***Barriers*** • Negative views about people living with HIV, such as reluctance to buy food from an HIV-positive vendor or support an HIV-positive teacher, were associated with lower testing rates • Low community acceptance of HIV-positive individuals hindered testing uptake. ***Facilitators*** • Knowledge of where to access testing services within the community increased the likelihood of testing. • Communities with greater media exposure demonstrated higher testing rates.
Yakasai & Yakasai (2022)	Nigeria	Cross-sectional	38,948	Women between 15 and 49 years in DHS	**Individual-Level Factors** ***Barriers*** • Low knowledge of HIV (only 33 % of respondents had good knowledge). • Negative attitudes toward HIV (75.9 % had a bad attitude). • Low perceived risk of HIV (95.8 % had a low perceived risk). ***Facilitators*** • Good knowledge of HIV significantly increased the likelihood of testing (OR = 3.81, *p* < 0.001). • Positive attitudes toward HIV increased testing likelihood (OR = 2.50, p < 0.001). • Medium and high perceived risk of HIV increased testing likelihood (Medium OR = 1.79, High OR = 2.03, p < 0.001). **Community-Level Factors** ***Barriers*** • Cultural beliefs about HIV, such as misconceptions regarding its transmission (e.g., through witchcraft), reduced testing likelihood (OR = 0.86, p < 0.001). ***Facilitator*** • Community education campaigns could counteract misinformation.
Okal et al. (2020)	Kenya	qualitative	38	HTC counsellors, men with HIV	**Individual-Level Factors** ***Barriers*** • Low perception of HIV risk • Fear of receiving a positive HIV test result • Limited knowledge or misconceptions about HIV transmission and management. ***Facilitators*** • High risk perception from engaging in risky behaviors • Severe illness motivating individuals to seek medical attention **Interpersonal-Level Factors** ***Barriers*** • Poor partner communication and lack of support for testing • Fear of disclosure and its impact on relationships ***Facilitators*** • Encouragement and support from partners, family, or close friends to undergo testing • Awareness of a partner's HIV-positive status **Institutional Level Factors** ***Barriers*** • Perceived negative attitudes of healthcare providers (e.g., unkindness, time-wasting) • Long wait times and inconvenient clinic hours conflicting with work schedules • Lack of privacy at health facilities • Insufficient counseling and quality of services ***Facilitators*** • Availability of counseling that helps clients cope with results and encourages linkage to care • Flexible operating hours to accommodate working men • Ensured confidentiality and discreet service setups. **Community Level Factors** ***Barriers*** • Anticipated stigma and discrimination from peers and community members • Negative stereotypes from peers and friends ***Facilitator*** • Community awareness campaigns addressing HIV stigma and promoting testing **Policy-Level Factors** ***Barrier*** • Cost of HIV testing

#### 3.1.1 Individual-level factors of HIV testing and counseling uptake

Sociodemographic predictors of HTC uptake from this review were age (high HTC uptake among women aged 15–24 years & men aged 25–34 years), and the number of children among women [23]. Also, the level of education, media exposure, number of lifetime sexual partners, household wealth index [23], and knowledge of HIV [23,24], increased the odds of HTC uptake. Further, HTC uptake was higher among Christians compared to other religious affiliations [23]. Being a rural residence, however, reduced the odds of HTC uptake [23].

Other facilitators of HTC uptake at the individual level included personal experience with HIV testing [14], privacy assurance using the self-test kit [14], ability to seek HTC [14,24], efficacy of HIV test [14] and ability to make time for HTC uptake [20], high HIV risk perception [[18], [19], [20], [21], [22],^24^], the possibility of having HIV [21,22] and severe illness [20]. Overall, the facilitators at the individual level were 51.28 % (Table 3).

**Table 3:** Frequency table showing the occurrence of the factors in the studies retrieved.

SEM Levels	Frequency (N)	Percentage (based on each SEM level) (%)
**Individual Level Factors**		
**Barriers**		
Poor knowledge of HIV transmission and prevention	3	7.69
Residing in rural areas	1	2.56
Lack of confidence in administering the test and interpreting results	2	5.13
Fear of making mistakes and obtaining false results	1	2.56
Distrust in the efficacy of oral self-tests (e.g., belief that saliva cannot detect HIV)	1	2.56
Preference for healthcare provider-led testing	1	2.56
Perception that self-testing is culturally or racially inappropriate	1	2.56
Lack of time to visit clinics for testing or picking up self-test kits	1	2.56
Fear of receiving an HIV-positive result and its associated stigma	3	7.69
Belief that HIV testing is unnecessary for those who feel healthy (Low risks perception)	3	7.69
Preference for self-testing (concerns about personal privacy and reluctance to disclose personal behaviors)	1	2.56
Desire for privacy and confidentiality in testing	1	2.56
Negative attitudes toward HIV	1	2.56
**Total Individual Level Barriers**	**20**	**51.28**

**Facilitators**		
Adequate HIV knowledge/higher educational level	2	5.13
Belonging to the higher wealth quintiles	1	2.56
High exposure to radio, television, and print media	1	2.56
Number of children ever born	1	2.56
Number of lifetime sexual partners	1	2.56
Belonging to the Christianity religion	1	2.56
Desire for privacy and avoidance of stigma associated with clinic-based testing	2	5.13
Perception of self-testing as time-saving and convenient	1	2.56
Prior experience with HIV testing enhances confidence in self-testing	1	2.56
Reduced fear of pain compared to blood tests	1	2.56
Increased perceived risk of HIV due to personal behaviors	3	7.69
Awareness of the importance of knowing HIV status for health management	1	2.56
Autonomy in managing one's health	1	2.56
Positive attitudes toward HIV increased testing likelihood	1	2.56
Severe illness motivating individuals to seek medical attention	1	2.56
**Total individual level facilitators**	**19**	**48.72**

**Interpersonal Level**		
**Barriers**		
Fear of partners and peer's reaction to test results, including violence or relationship breakdown	2	13.33
Concerns about partner trust	2	13.33
Fear of unintentional disclosure to partners, peers, or family members	2	13.33
Poor partner communication and lack of support for testing	1	6.67
**Total interpersonal level barriers**	**7**	**46.67**

**Facilitators**		
Willingness to test with partners to learn each other's status	2	13.33
Support from partners and peers to pursue testing and follow-up care	3	20.00
Open communication with partners and family members about HIV testing	1	6.67
Peer acceptance and normalization of testing behavior	2	13.33
**Total interpersonal level facilitators**	**8**	**53.33**

**Institutional Level Factors**		
**Barriers**		
Concerns about privacy during clinic visits	3	13.04
Stigma at the clinic settings	2	8.69
Perceived judgmental attitudes of healthcare workers	2	8.69
Absence of post-test counseling (Insufficient counseling and quality of services)	2	8.69
Limited accessibility to HIV testing services in rural areas	1	4.35
Low availability of information about testing locations	1	4.35
Long wait times and inconvenient clinic hours conflicting with work schedules	1	4.35
**Total institutional level barriers**	**12**	**52.17**

**Facilitators**		
Positive past experiences and attitude of healthcare providers, including supportive and respectful interactions	2	8.69
Counseling services at health facilities	3	13.04
Trust and confidence in healthcare workers' and facilities	1	4.35
Health facilities are free of discrimination	2	8.69
Integration of HIV testing into antenatal care for women increased testing uptake	1	4.35
Flexible operating hours to accommodate working men	1	4.35
Ensured confidentiality and discreet service setups	1	4.35
**Total institutional level facilitators**	**11**	**47.82**

**Community Level Factors**		
**Barriers**		
Stigma and discrimination from peers and community members	3	20.00
Misconceptions and stereotypes about individuals who seek HIV testing, e.g., assumptions of immoral behavior	5	33.33
**Total community level barriers**	**8**	**53.33**

**Facilitators**		
Community-based education programs to reduce stigma and promote testing	5	33.33
Outreach programs to raise awareness about HIV prevention and testing services	1	6.67
Communities with greater media exposure demonstrated higher testing rates	1	6.67
**Total community level facilitators**	**7**	**46.67**

**Policy Level factors**		
**Barriers**		
High cost of self-test kits (perceived as unaffordable by participants)	3	42.86
Lack of incentives for HIV testing	1	14.29
**Total policy level barriers**	**4**	**57.14**

**Facilitators**		
Availability of HIV self-testing for improved privacy	2	28.57
Availability of HIV tests at both public and private facilities	1	14.29
**Total policy level facilitators**	**3**	**42.86**

Barriers at the individual level included discomfort with HIV tests [14,21], fear of HIV test results [[20], [21], [22]], preference for self-test [21], low HIV risk perception (HIV test is unnecessary) [20,21], no time for HTC uptake [21], and limited HIV knowledge [20]. Overall, individual barriers aggregated to 48.72 % (Table 3).

#### 3.1.2 Interpersonal-level factors of HIV testing and counseling uptake

Enablers of HTC uptake at the interpersonal level included discussing HTC uptake with family and sexual partners [14,20,21], testing with sexual partners [14,21], distrust of partners fidelity [14], and willingness to encourage HIV-positive partner to seek care while practicing safe sex [14]. Also, support and positive attitude of peers, friends, and family [21], severe illness [20], and awareness of partner's status [20] increased HTC uptake. Overall, the interpersonal level enablers of HTC aggregated to 46.67 % as shown in Table 3. The barriers, on the other hand, were concerns about partners' reactions in case of discordant HIV results [14,[20], [21], [22]], and minimal discussions about HTC [20]. From Table 3, the interpersonal level barriers of HTC aggregated to 53.33 %.

#### 3.1.3 Institutional-level factors of HIV testing and counseling (health system)

Institutional level facilitators of HTC uptake included positive experiences with healthcare providers [14,21], usefulness of HIV test [21], no discrimination at the health facility and integration into antenatal care services [14,23], confidence in health facilities [14], provision of counseling and support about HIV risk behaviors [14,20,21], confidentiality assurance [21], and extended/flexible health facility opening hours [20]. The institutional level facilitators aggregated to 47.82 % (Table 3).

Institutional factors that inhibit HTC uptake as obtained from this review include lack of privacy and confidentiality at the health facility [14,[20], [21], [22]], stigma at clinic setting [14], the attitude of healthcare providers [20,21], and prolonged biomedical protocol before results are obtained [22]. Also, distance from the health facility, inconvenient clinic times, and the quality of services and supplies hindered HTC uptake [20]. The barriers at the institutional aggregated to 52.17 % (Table 3).

#### 3.1.4 Community-level factors of HIV testing and counseling uptake

Community level facilitators of HIV testing included community awareness of voluntary testing and counseling services [[20], [21], [22], [23]], campaigns to reduce stigma and promote testing [21], and media influence as communities with high media exposure [23] reported higher rates of HIV testing. Overall, the facilitators of HTC at the community level aggregated to 46.67 % (Table 3). Further, from the review, adverse social norms about HIV hinder HTC uptake [22]. Also, Yakasai and Yakasai [24] reported cultural beliefs about HIV, such as misconceptions regarding its transmission (e.g., through witchcraft), reduced HTC uptake. In addition, stigma and discrimination impede HTC uptake [20]. Further Okal et al. [20] and Mendy et al. [21] reported that negative stereotypes from peers or friends were barriers to HTC uptake. These together aggregated to 53.33 % (Table 3).

#### 3.1.5 Policy-level factors of HIV testing and counseling uptake

Inhibitors of HTC uptake at the policy level from the review included the cost of testing [14,20,22], and lack of incentives for HIV testing [14]. The barriers aggregated to 57.14 % as indicated in Table 2. In addition, the availability of HIV self-testing for improved privacy (Tanzania) [22], and the availability of HIV tests at both public and private facilities [14] were identified as policy-level facilitators to improve HTC uptake. These aggregated to 42.86 % (Table 3).

## 4 Discussion

This systematic review highlights the multilevel factors influencing HTC uptake among adults in SSA using the SEM. Individual-level factors were most frequently reported, with education, HIV knowledge, perceived risk, and prior testing experience facilitating uptake, while fear, low perceived risk, and limited knowledge acted as barriers [14,[20], [21], [22], [23], [24]]. At the interpersonal level, partner communication and support encouraged testing, though fear of discordant results and minimal discussions hindered uptake [13,[18], [19], [20]]. Institutional facilitators included confidentiality, provider support, and service accessibility, while stigma, lack of privacy, and provider attitudes posed significant challenges [14,[20], [21], [22], [23]]. Community awareness and media exposure promoted testing, yet stigma and cultural misconceptions remained barriers [[20], [21], [22], [23], [24]]. At the policy level, cost and lack of incentives limited access, although the availability of self-testing and services across facilities showed potential to increase HTC uptake [13,20,22]. Overall, HTC uptake is shaped by intersecting influences across SEM levels, underscoring the need for comprehensive, context-specific strategies to improve HTC uptake, especially among underserved populations.

A key strength of this review is its theory-driven synthesis using the SEM, which provided a structured lens to identify intervention entry points at different levels [[9], [10], [11]]. Contrary to prior reviews that did not use guiding framework [[25], [26], [27]], our review illustrates how facilitators and barriers are distributed across the SEM and where interventions can be targeted. However, including only studies that explicitly applied the SEM may have led to the exclusion of studies with relevant findings aligned to SEM constructs. Also, most of the included studies were cross-sectional or qualitative in design, and therefore primarily assessed immediate or short-term factors influencing HTC uptake. This limits our ability to infer long-term outcomes such as sustained behavior change or retention in care. Furthermore, while this review synthesizes studies from various countries in SSA, the transferability of findings may be limited due to contextual differences across regions, particularly among underserved populations. Factors such as healthcare infrastructure, cultural norms, and socioeconomic disparities may influence the applicability of these results in other SSA settings or marginalized communities.

The findings from this review are consistent with earlier studies across SSA that highlight the central role of education and HIV knowledge in promoting HTC uptake [25,[28], [29], [30], [31]]. Also, the importance of support from peers, partners, and family members align with previous studies in SSA and Kenya, which highlight how social support can reduce stigma and increase willingness to test [25,27,32]. However, contrasting evidence from Ghana suggests that some youth prioritize privacy over support, avoiding HTC discussions with family and peers [33]. The fear of negative partner reactions in cases of discordant results, with potential risks of emotional or physical abuse including minimal communication about testing within social networks aligns with findings from Ghana, where individuals living with friends were less likely to undergo testing [28]. These results highlight how interpersonal dynamics can both enable and hinder HTC uptake, depending on social context and relational trust. Similarly, fear of stigma and lack of confidentiality in healthcare settings have repeatedly been shown to deter individuals from testing, particularly in settings with limited privacy safeguards [[25], [26], [27],^34^,^35^].

This review extends prior work by showing that these factors operate not in isolation but across multiple SEM levels. For instance, individual fear is often reinforced by community-level stigma and institutional-level breaches in confidentiality. While previous reviews have focused predominantly on individual and health system barriers [25,26], our findings suggest that interpersonal and policy-level influences such as partner dynamics and the cost of testing are also elements to consider when planning and implementing targeted intervention for HTC uptake.

## 5 Conclusion

This review offers a structured synthesis of the multilevel facilitators and barriers to HTC uptake among adults in SSA, guided by the SEM. By mapping findings across individual, interpersonal, institutional, community, and policy levels, it highlights the complex and intersecting determinants that shape HTC behavior in the region.

### 5.1 Implications for practice

Interventions to improve HTC uptake must be multi-level and context-specific, addressing both personal and structural barriers. Strategies should include expanding HIV education to increase knowledge and risk perception, strengthening confidentiality and provider-patient trust within healthcare settings, reducing community-level stigma, and eliminating cost-related barriers to testing. A holistic approach that integrates these components is essential for advancing equity, expanding access to HIV testing and treatment, eliminating HIV-related stigma and achieving UNAIDS 95–95-95 goals and the WHO Global Health Sector Strategy on HIV by 2030.

### 5.2 Implications for research

Future research should prioritize longitudinal and mixed-methods studies to better understand how individual and structural factors interact over time to influence HTC uptake. There is also a need to explore underreported domains, particularly policy-level facilitators, and to examine intervention effectiveness across different population subgroups. Expanding the evidence base in these areas will support the development of targeted, sustainable strategies for increasing HTC coverage in SSA. Further, future reviews may consider incorporating studies that address multi-level factors consistent with the SEM, even if the model is not explicitly stated, using structured deductive coding frameworks to ensure conceptual alignment.

## Authors contribution

**ADD**: selection of articles, quality assessment of articles, data extraction, drafting of initial manuscript, review of the final version of the manuscript. **EET:** selection of articles, quality assessment of articles, data extraction, drafting of initial manuscript, review of the final version of the manuscript. Both authors read and approved the final manuscript.

## CRediT authorship contribution statement

**Amanda Debuo Der:** Writing – review & editing, Writing – original draft, Visualization, Validation, Resources, Methodology, Formal analysis, Data curation, Conceptualization. **Elvis Enowbeyang Tarkang:** Writing – review & editing, Validation, Supervision, Resources, Methodology, Data curation, Conceptualization.

## Consent for publication

N/A.

## Ethical approval and consent to participate

N/A.

## Funding

This research project was funded by the authors.

## Declaration of competing interest

The authors declare that they have no known competing financial interests or personal relationships that could have appeared to influence the work reported in this paper.

## Data Availability

All articles included in the final review have been carefully cited.

## Appendix A Supplementary data

Supplementary material 1 PRISMA flowchart showing the process of the review Image 1
