# Supplementary material for: Socio-ecological model: Understanding facilitators and barriers to HIV testing and counseling uptake in sub-Saharan Africa – A systematic review
Source: Dialogues Health. 2025 Dec 23;8:100273. doi: 10.1016/j.dialog.2025.100273 (PMC12813316; doi:10.1016/j.dialog.2025.100273)
Supplement: Supplementary file 1 — Supplementary material 1 PRISMA flowchart showing the process of the review [file mmc1.docx]

**Identification of studies via databases and registers**

Records removed *before screening*:

Duplicate records removed (n = 31)

Records identified from*:

Databases (n = 114)

**Identification**

Records screened

(n = 83)

Records excluded**

(n = 2)

Reports sought for retrieval

(n = 81)

Reports not retrieved

(n = 0)

**Screening**

Reports assessed for eligibility

(n = 81)

Full text articles excluded with reason (n= 75)

-Not on HTC uptake (n = 23)

-Conducted outside SSA (n = 10)

-Did not use the SEM (n = 40)

Studies included in review

(n = 6)

**Included**

Source: Page MJ, et al. BMJ 2021;372:n71. doi: 10.1136/bmj.n71.

This work is licensed under CC BY 4.0. To view a copy of this license, visit <https://creativecommons.org/licenses/by/4.0/>
